# Perspectives from clinicians from different levels of care in Maputo, Mozambique: qualitative study of the barriers to and facilitators of paediatric injury care in resource-poor hospital settings

**DOI:** 10.1136/bmjopen-2024-085270

**Published:** 2024-11-24

**Authors:** Vanda Amado, Americo Zandamela, Maria Tereza Couto, Lee A Wallis, Lucie Laflamme

**Affiliations:** 1 Department of Global Public Health, Karolinska Institute, Stockholm, Sweden; 2 Faculty of Medicine, Eduardo Mondlane University, Maputo, Mozambique; 3 Marracuene, INS, Maputo, Mozambique; 4 University of Cape Town, Rondebosch, Western Cape, South Africa; 5 Karolinska Institute, Stockholm, Sweden; 6 University of South Africa Institute for Social and Health Sciences, Pretoria, Gauteng, South Africa

**Keywords:** Resource-poor setting, Trauma care, Tertiary hospitals, Pre-hospital care, Child injury, Injury mortality and morbidity, Barriers to injury care, Mentorship

## Abstract

**Abstract:**

**Objectives:**

Providing care for injured children is challenging in resource-poor settings. While checklists can assess local capacities and guide the setting of priorities for improvement, key insights can be gained from consultation with locally practising clinicians. This study aimed to highlight barriers to and facilitators of the delivery of paediatric injury care experienced by clinicians from hospitals at different levels of care in Maputo, Mozambique.

**Design:**

We conducted semistructured individual qualitative interviews with clinical staff at four hospitals. Data were analysed using inductive content analysis.

**Setting:**

The study was conducted in four hospitals, each representing a specific level of care in Maputo, Mozambique.

**Participants:**

We recruited clinicians (doctors, nurses and technicians) involved in paediatric injury care to be interviewed on-site (we target around 10 clinicians per hospital).

**Results:**

From the 40 interviews conducted, four categories of barriers emerged: (1) prehospital care constraints, (2) shortage of child-appropriate resources, (3) inappropriate infrastructure for paediatric emergency care and (4) limited qualified staff available. By contrast, one category of facilitators stood out, namely that of cross-boundaries support and mentorship, between professionals and institutions.

**Conclusion:**

From clinicians’ perspective, barriers to paediatric injury care are often similar across hospitals and professional groups, and they include the prehospital setting. Resource and infrastructure challenges were emphasized, as expected, and clinicians expressed a clear desire for knowledge and competence sharing.

**STRENGTHS AND LIMITATIONS OF THIS STUDY**

- The study includes clinicians representing different professional groups from all levels of care.
- The focus was on paediatric injury care, seeking barriers and facilitators within and outside the hospital setting.
- As the interviews were conducted in Maputo province, which has relatively better healthcare resources, the results may not be fully transferable to other settings in the country.
- The interviews were conducted privately but at work and during working hours, which may have put the interviewees under time pressure/stress.
- The data collectors were included in the coding of the interviews, but no informant checks took place.

## Introduction

 Globally, injuries are the leading cause of death among children aged 5 to 14 years,^1 2^ and 95% of those deaths occur in low- and middle-income countries (LMICs).^2^,^4^Injuries are also the largest source of premature morbidity and mortality globally, regardless of age, sex, income or geographic region.^1 5 6^ Timely quality healthcare can significantly improve poor injury outcomes by ensuring the provision of essential emergency care services by well-trained staff.^7^,^9^ This need is particularly clear in resource-poor settings,^2 10^ especially Sub-Saharan Africa (SSA),^11 12^ where prehospital emergency response is generally underdeveloped^13^ and hospital emergency services suffer from shortages in staff, technical resources and medications for injury care.^14 15^

An additional set of constraints in the case of injured children is that they are typically treated without adequate regard for their specific needs^16^,^18^ by clinical staff ill-prepared or unfamiliar with paediatric injury care.^19 20^ This, in turn, increases the likelihood of diagnostic error and inadequate treatment with the potential of worsened outcomes and low survival rates.^18^

Another common finding in SSA is the quality gap between public and private hospitals, documented among others in South Africa, Uganda, Kenya and Rwanda, to the detriment of public hospitals.^21^ This is particularly the case at first-level (district) hospitals where critical care services are either non-existent or very resource-constrained.^21^

SSA studies investigating hospital care services typically focus on hospitals from a specific level of care across the country^22^ or, in some instances, on hospitals from several levels of care.^23 24^ A focus group with medical providers in a referral hospital in Uganda, for example, identified major barriers to caring for critically ill children including limited resources, staffing and training gaps.^22^ A study looking at all 30 primary and secondary hospitals in western Kenya found that the provision of core emergency care services for injuries and non-communicable diseases suffered from a range of issues including gaps in communication, infrastructure, supplies and trained human resources.^23^ In the Kingdom of Eswatini, a study evaluating emergency care capacity in three referral hospitals highlighted concerns related to infrastructure barriers for critical care (eg, inadequate physical space, electricity or water) and a lack of equipment, personnel knowledge and skills.^25^

In Mozambique, a recent study on paediatric injury care in the country’s four largest referral hospitals^26^ found that these hospitals had significant shortages of medications (eg, for infections, poisoning and cardiovascular disorders) and certain equipment (eg, for diagnosis and monitoring, patient safety and airway management). The extent of these shortages in hospitals at other levels of care in the country has not been documented, which leaves unanswered questions regarding care provision at these sites. The perception of the staff in those settings is also not known but is something that could give better insight into how problems are experienced and dealt with locally, what solutions are envisaged and whether there is consensus in those aspects. This study therefore explores how barriers to and facilitators of paediatric injury care at different levels of care are described by healthcare staff. It focuses on the context of Maputo, Mozambique, where the main referral hospital of the country is located and where several hospitals at different care levels are also located.

## Methods

### Study design

We conducted a qualitative interview-based study using one-to-one structured interviews with healthcare providers who take care of injured children to explore the barriers to and facilitators of better paediatric injury care. Inductive content analysis was used to better elicit the narrative of the health professionals.^27 28^

### Setting and selection of hospitals

**Figure 1:**
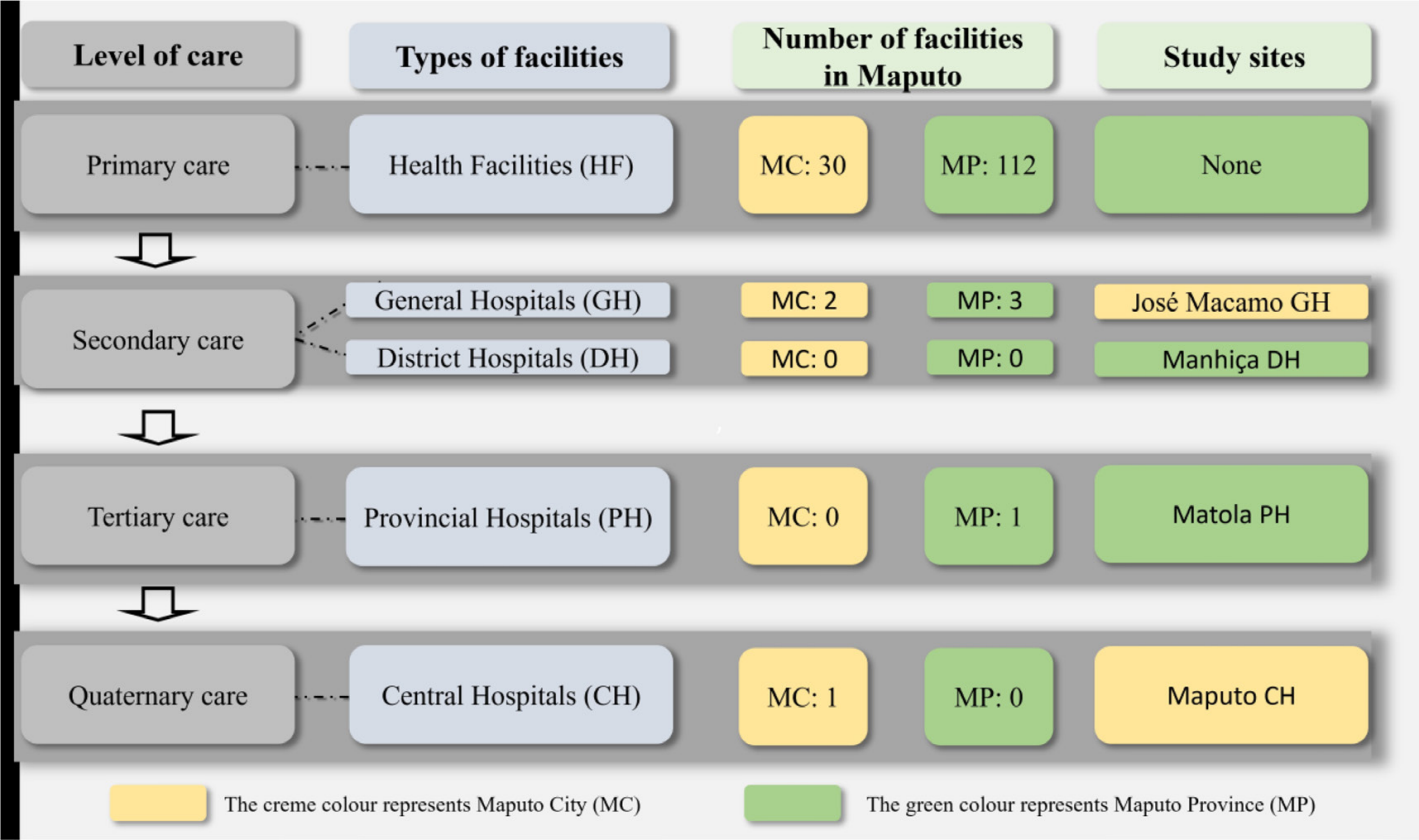
Healthcare system organisation in Maputo and selection of hospitals for the study. Author created (Vanda Amado).

In this study, a mix of all levels of care including district and referral hospitals was chosen, focused on Maputo, the capital and the country’s biggest city. From Maputo city, we chose Maputo Central Hospital, the only central one in the country—and one of the two secondary hospitals (Jose Macamo General Hospital), located in the suburbs of Maputo city. From outside the city but still in the province, we chose one tertiary hospital (Matola Provincial Hospital) and one secondary hospital (Manhiça Distrital Hospital), respectively, 18 and 82 km from Maputo Central Hospital (see figure 1).

Mozambique is a low-income SSA country with an estimated 60% of the 33.7 million inhabitants (2023 data) living in rural areas with no or limited access to healthcare.^29^ The country is divided into 10 provinces and one capital city, Maputo, with provincial status. Overall, Mozambique has around 1600 health facilities, 96% of which only deliver primary care services.^30^

Most of the population has access to free public healthcare services, organised into four levels: primary care (health centres), secondary care (rural hospitals; district or general hospitals), tertiary care (provincial hospitals) and quaternary care (central hospitals). Mozambique does not have any trauma centre, paediatric hospital or trauma system organisation.^31 32^

### Participant selection and sample size

Healthcare workers were purposively selected from Maputo Central Hospital, Jose Macamo General Hospital, Matola Provincial Hospital and Manhiça Distrital Hospital.^28 33^

The healthcare workers were chosen to represent those with a range of knowledge and experience in paediatric injury care. They would contribute to identifying and obtaining information related to barriers to and facilitators of better paediatric injury care.

During the visit, clinicians taking care of injured children and working full time were asked to participate in an interview. We sought clinicians of different cadres, including surgery technicians, nurses and medical doctors from different categories such as general practitioners; surgery residents; paediatricians; and general, orthopaedic, neuro, plastic and paediatric surgeons.

We aimed to interview around 10 clinicians per hospital, covering the most representative professional categories (surgeons, paediatricians, nurses and technicians). We proceeded until we felt thematic saturation was reached,^34^ and additional interviews would not generate new themes.^35 36^

None of the clinicians contacted refused participation or dropped out of the interview.

### Data collection

A semistructured interview guide was developed in Portuguese that incorporated the areas of concern highlighted in our earlier study conducted in the central hospital.^26^ The interview guide used in this study is provided as online supplemental annex 1. The clinicians were asked about the main difficulties they encountered while taking care of injured children, why they thought these difficulties existed and in what way they impeded quality care. They also answered questions on how they cope with the difficulties, what the main facilitators of injury care could be and how to move forward to make improvements.

The data collection involved three people, the principal investigator (PI) and two experienced research assistants (RAs), educated in social anthropology and psychology, who received specific training for the study. The RAs performed the interviews under the coordination of the PI who also recruited the interviewees. Data collection took place over four consecutive weeks, during working hours.

Before the interviews, the RA obtained written consent. All those contacted gave their consent. All interviews were face-to-face and performed in a quiet meeting room at the hospital, at a time most convenient for the interviewee. The interviews were audio-recorded for transcription, and field notes were taken. No repeat interviews were carried out. For each hospital, the PI and the two RAs discussed data saturation. The duration of the interviews varied from 35 to 60 min. No transcriptions were returned to participants for comments or correction.

All material was kept anonymous. The audio recordings were transferred to a password-protected computer and stored, while the original recording was deleted. All consent forms and de-identified transcripts were stored in a protected locker belonging to the PI and first author.

### Ethical considerations

In June 2022, the management of each targeted hospital received a request letter for participation in the study, which they all accepted. With their acceptance letter, the study protocol was submitted for ethical approval by the Mozambique Institutional Bioethics Committee of the Faculty of Medicine and Maputo Central Hospital (CIBS FM & HCM/107/2019). Once ethical approval was obtained, the director of each hospital was contacted to suggest a time suitable for data collection. The directors informed their staff about the visit, and voluntary participation was emphasised.

### Observations

To get familiarised with the work environment of each hospital and contextualise capture the barriers to and facilitators of paediatric injury care described by the people interviewed, the PI and RAs observed for 8 hours split in two weekdays. They observed the infrastructure, equipment in place, specialised injury care delivery and how the health workers performed their tasks. Note was taken of anything related to the premises, equipment and tasks, or task division that was felt as susceptible to complicate the execution of the tasks or, rather, facilitate trauma care activities^37^ was noted.

### Research team and reflexivity

The research team and authors include three women and two men with different levels of seniority. Two of the women are specialised surgeons from Maputo, and the third one is a senior scientist specialised in injury epidemiology on prevention studies, including in the clinical field. One of the men is an anthropologist with experience in qualitative data collection in Mozambique, and the other is a medical doctor specialised in emergency medicine with considerable international experience.

This paper was an attempt to shed light on the barriers to and facilitators of paediatric trauma care experienced by clinical staff at different levels of care. The data collection was led by a paediatric surgeon—and doctoral student—very familiar with the Mozambican context, assisted by two experienced data collectors who conducted all the interviews. The recruitment of interviewees went well, and the interviewers were well-prepared in order to establish a relationship of trust before engaging in the interviews. The data analysis (that of the interview transcripts) took place in different steps, involving first to those who were directly involved in the data collection, all native Portuguese speakers who immersed themselves in the coding process. Other co-authors were involved in discussions about the subcategories and categories.

### Patient and public involvement

Neither injured patients nor the public were involved in the study design, data collection, recruitment, conduct or analysis of this study.

The research questions and outcomes were based on a gap in the literature related to how the shortage of equipment and medications found in the country’s four largest referral hospitals^26^ affect other levels of care. They also took into account the perception of the healthcare providers on the barriers to and facilitators of paediatric injury care. We hope the results will inform future researchers and help implementation researchers improve paediatric injury care in LMICs. We also plan to disseminate the information at national and international conferences.

### Data analysis

All interviews were recorded and transcribed *verbatim* in Portuguese, while the study team members also took written notes in case there were problems with recordings. Transcripts were coded in NVivo, V.14.^38^ The first author and one RA coded the data, starting with familiarising themselves with and reviewing the transcriptions while listening to the audio recordings several times. Meaning units were inductively identified and translated to codes. These were then revised, organised and compared for consistency, and discrepancies were resolved among the researchers. The codes were thereafter classified into subcategories and categories based on their similarities and differences. Results were written after re-reading the categories and subcategories.

## Results

### Characteristics of the participants

A total of 40 participants were interviewed. Their characteristics are shown in table 1. The average age varied between hospitals, from 31 years in Manhiça Distrital Hospital to 42 years in Matola Provincial Hospital. Most participants were male (n=26). Nurses were the most represented profession (62.5%), and the average length of years employed ranged from 5 to 8 years.

**Table 1:** Study participant’s characteristics

Characteristic	Maputo Central Hospital	Jose Macamo General Hospital	Matola Provincial Hospital	Manhiça Distrital Hospital
	n=9	n=9	n=11	n=11
	N	N	N	N
Sex				
Male	3	8	8	7
Female	6	1	3	4
Employment				
Doctor/specialist	4	4	4	3
Nurse/technician	5	5	7	8
	Average (range)	Average (range)	Average (range)	Average (range)
Employment (in years)	5 (4–8)	8 (7–9)	7 (4–14)	5 (2–9)
Age (in years)	39 (26–64)	37 (33–49)	42 (36–50)	31 (27–40)

### Barriers

Before moving on to the barriers, we highlighted the resources that the clinicians most reported as missing in each hospital, as these either impeded the provision of quality care on-site or necessitated sending patients to a higher level of care. Those missing resources are summarised in table 2, ordered from the hospital at the highest level of care to the lowest, and classified into competence, equipment, paediatric intensive care (PICU) and specialised care.

**Table 2:** Hospital-specific capacity limitations reported by the interviewees, from the quaternary hospital to secondary care

Resources	Level of paediatric care/selected hospitals			
	Quaternary	Tertiary	Secondary	Primary-secondary
	Maputo Central Hospital	Matola Provincial Hospital	Jose Macamo General Hospital	Manhiça Distrital Hospital
Specialised clinical staff				
Paediatric surgeon	X			
Neurosurgeon	X			
Paediatric orthopaedic	X			
Paediatric emergency	X	X	X	
General surgeon	X			
General paediatric	X	X	X	
General orthopaedic		X	X	X
Surgical technician	X	X	X	X
General medical doctor				
EquipmentCAT, *MRI*, sonography,monitoring device	Yes, but not in paediatric emergency	None	None	None
PICU	Yes, but not attached to the operating theatre	None	None	None

CATcomputed axial tomographyPICUpaediatric intensive care unit

From the narrative of the participants, four main categories of barriers emerged (see table 3), labelled prehospital care constraints, shortage and limitation in child adaptability of resources, inappropriate infrastructure for emergency care and limited staff.

**Table 3:** Four categories of barriers emerged from the interviews and related subcategories

Categories	Subcategories
Prehospital care constraints	Access to the nearest hospitalCaregiver’s consent to child transferReferral to a higher level of care
Shortage in child adaptability of resources	Medication and medical supplies
	Diagnosis and monitoring equipment
Inappropriate infrastructure for paediatric emergency care	Space: small spaces, shared place with adults, few bedsResources not available: PICU, emergency operating theatre
Limited qualified staff available	Qualifications and training of staff
	A limited number of clinical staff

PICUpaediatric intensive care unit

### Prehospital care constraints

Clinicians from all hospitals reported as a main barrier the difficulties of transporting children from the injury site to the hospital most suited for their care, largely due to traffic jams, quality of the roads and frequent road traffic crashes.

*Access to the nearest hospital*. Most of the clinicians talked about the frequency of road traffic injuries and the fact that several road traffic crashes involve overcrowded public transport vehicles. Those crashes lead to serious traffic jams and obstruct the transportation of victims to hospital. Injured children are often referred to the nearest hospital using private cars or evacuated in police cars without any care, arriving at hospital in a worse condition and with secondary injuries.

*We do not have prehospital care in which there are doctors who take care of injured patients at the scene of the accident…It takes a very long time to arrive at the Maputo Central Hospital, some arrive in a very critical condition that is perhaps impossible to recover from.* (Doctor 1, Maputo Central Hospital)

*Caregiver’s consent*. After the first assessment by the team, critical patients must be referred to a specialised hospital to continue the treatment, but generally, caregivers—mainly mothers—hesitate to give their consent either for financial concerns, because one parent would like to have the assent of the other or because they have other children to take care of.

*There are situations in which…we take a while to refer because the decision is not made by the person who is the hospital…the mother just says that the father is the one who can decide, the father is travelling…and he will arrive in a few hours. So, it makes us waste precious time*. (Nurse 1, Manhiça Distrital Hospital)

*Referral*. Clinicians also raised issues about the low number of ambulances available, which delays the initiation of care. Besides being few, those ambulances are poorly equipped for injury care. Injured patients compete with other emergencies and priorities. In the referral of patients, the vehicles often do not have oxygen, monitoring devices or ambulance stretchers, so a nurse must escort the patient. This reduces the number of staff available at the hospital, and, when the transport takes time, the patient may die during transport.

*Sometimes, we want to refer the patient in time, but the ambulance is at the Maputo Central Hospital because it was transporting another patient. When the injured child arrived, we stabilised, but we must wait for the ambulance to return.* (Doctor 2, Matola Provincial Hospital)

### Shortage in child adaptability of resources

*Medication and medical supplies*. Lack of medication and medical supplies was mentioned as affecting the quality of care and imposing limitations on interventions, making the work of the providers more complex. The lack of specific supplies for paediatric care such as splints, cervical collars, suitable sutures and catheters for children’s veins results in improvisation, compromising the effectiveness of procedures. Furthermore, it was mentioned that the lack of resources could affect patient safety and the quality of interventions.

*They are observed in the same place where adults are observed, and the same instruments are used. This is where the problem begins, the device for measuring blood pressure in adults is large and it will not be possible to measure blood pressure in a child when they enter intensive care services for adults too, if we want to insert an orotracheal tube then, the material used to insert the tube is for adults, it is not paediatric, so this leaves the child at a disadvantage*. (Doctor 3, Maputo Central Hospital)

*Equipment*. A lack of diagnosis and monitoring devices as well as anaesthesia machines adapted for paediatric patients was often reported by professionals from all level health facilities as a very common challenge for paediatric injury care and a quality deterrent. A shortage of CT scans, X-rays and anaesthesia machines limits the clinicians in performing diagnostic tests, operating in well-adapted rooms and monitoring injured victims in a critical condition.

*…Computerised axial tomography (CAT)…is a prior need to carry out some tests to identify the damage in detail, so we always choose to refer when it comes to moderate and severe traumatic brain injuries…because the Maputo Central Hospital is the only health unit that, until now, had CAT scans available*. (Doctor 4, Matola Provincial Hospital)

### Inappropriate infrastructure for emergency care

Respondents from all hospitals reported that the emergency department is inadequate for paediatric injury care as there is no specific place for injured patients: all emergency patients are admitted to the adult emergency department, which is not necessarily equipped or suitable for paediatric emergency care.

*Spaces*. Clinicians mentioned that the small dimensions of the rooms where they treat injured patients obstruct the circulation of the staff and threaten the privacy of the patient (and family). The lack of space separators also limits the number of available beds (to about four to eight) when far more would be needed. So, injured children are cared for by adult clinicians, not in child-friendly spaces, mixed with adults of different sexes and with patients with other emergency conditions like malaria or tuberculosis.

*It is not an emergency service specific to trauma care. It is an emergency service that also treats other pathologies. So, you’re there with an injured child and with a patient with tuberculosis, COVID, e.g.…maybe the injured child runs the risk of being contaminated with other diseases, this is the risk we have because we don’t have an infrastructure for handling trauma in general*. (Nurse 2, Matola Provincial Hospital)

*Resources*. In all hospitals, respondents reported a lack of adequate facilities for paediatric injury care, with some differences based on the level of care of the hospital (see also table 2). In secondary and tertiary health facilities, clinicians reported a lack of PICU as one of the main reasons for referring critically injured patients to quaternary health facilities. They also mentioned that the emergency facility is unable to stabilise critically injured patients while waiting for the ambulance due to lack of oxygen, monitoring devices and fluids in this sector.

*…because we do not have an intensive care unit, or specialists to care for severe head trauma, we transfer these patients*. (Nurse 3, Manhiça Distrital Hospital)

PICU is available in the quaternary hospital, although clinicians mentioned that they do not have attached paediatric operating theatres, access to diagnostic tests like CT or even permanent surgeons to care for the injured child. An injured child is first admitted to the adult emergency by general surgeons, which results in errors in care and, when combined with a lack of access to diagnostic tests, affects the outcome. Patients are often sent from the PICU back to adult emergency for diagnostic testing, but an ambulance is required for this transfer which leads to significant delays in care.

*For example, if there is a traumatic brain injury, it should be evaluated by a neurosurgeon in the adult’s emergency to facilitate the diagnosis, treatment, and monitoring, before the injured child is referred to PICU. Sometimes the injured child comes without imaging exams and no care for her critical condition. Due to her critical situation, the child is not sent back to the imaging examination. So, one of our problems is that we don’t have, for example, the CAT, close to our intensive care unit and we must stabilise critical patients blinded without the auxiliary diagnosis*. (Doctor 5, Matola Provincial Hospital)

### Limited staff available

*Qualifications*. The paucity of staff well-trained to deal with paediatric injury patients was highlighted by clinicians from all four hospitals as was the lack of specialised clinicians (eg, paediatric surgeons, anaesthetists orthopaedics). Some health professionals reported that their previous education and experience often focused on adult care, making the transition to paediatric care difficult.

*Unfortunately, we had never had any paediatric training, capacity building or refresher training, we are limited, doing what we have learned at school.* (Nurse 4, Manhiça Distrital Hospital)

*Staff*. Most respondents underlined staff shortages and lack of staff training as serious challenges to quality paediatric injury care. They also report that because of the few radiologists available, they cannot do 24-hour imaging exams and in secondary care, and no X-rays are available after 3:30 pm and during the weekends.

*The first major limitation we have is the technical personnel, the personnel prepared to work with paediatric injury. Now we only have two surgical technicians who are working in the trauma section, and they…don’t work at the same time, one period is one…From morning until 1400 there is one, from 1400 to 2000 there is another. So, there are only two of them monitoring, so it is a huge overload for them and this huge overload ends up leading to a loss of efficiency, people with a lot of work like this are not able to be completely attentive all the time, the concern is to care in a short time the largest number of patients and it will not be based on quality, we are mainly based on the number of patients we serve.* (Doctor 6, Jose Macamo General Hospital)

### Facilitators

From the health professionals’ narrative, one main category of facilitator emerged: support and mentorship between professionals and institutions (see table 4).

**Table 4:** One category of facilitator emerging from the interviews and its subcategories

Categories	Subcategories
Support and mentorship, between professionals and institutions	Mentorship of qualified team
	Collaboration of quaternary care
	Support of local and international institutions

*Mentorship of a qualified team*. Participants reported that the emergency team contributes to facilitating the care of paediatric injuries in different ways. Both surgeons and surgical technicians gave great support to the team in serious and complex trauma situations, filling gaps and overcoming shortcomings. This cooperative environment avoids overloading health professionals and helps coordinate activities resulting in an improvement in the quality of care offered to injured children and in the solution of problems and challenges faced. The presence of a diverse and highly qualified team and the appropriate number of teams per shift facilitates injury paediatric care and allows for the exchange of experience.

*Collaboration of quaternary care*. Participants from secondary and tertiary care have the opinion that Maputo Central Hospital facilitates their work. Communication via WhatsApp and video calls helps coordinate the referral process and allows the support of specialised clinicians with clinical advice to stabilise and manage critical patients, facilitate injury paediatric care, and exchange experience and resources.

*One of the facilitators is the good collaboration and relationship that we have, with the Maputo central hospital…Through phone consultation with paediatric surgeons for difficult cases and support…I think this good relationship is also one of the facilitators…* (Doctor 7, Hospital Provincial da Matola)

Most specialists from quaternary care consider paediatric emergency care and PICU as one of the most important facilitators for paediatric injury care as they stabilise and optimise the critical injury for further care or after surgery.

*When a severe traumatic brain injury child is admitted in the paediatric emergency department…it makes our work tremendously easier because as soon as the child arrives, s/he receives all the care that is set out in our protocol and paediatrics protocols in place…so when I receive the call to attend in the paediatric emergency, I go knowing that the child has received first aid is stabilised, they call me I will be more cheerful…whereas if I know that they are calling me for a child in the adult emergency room, I will already be very stressed, why? I’m scared of what I’m going to find. So, I would say that the paediatric emergency service is a great facilitator in our care for traumatic brain injury, when the child arrives, when we take the child to the operating room and return them to paediatric intensive care, ha! The care provided post-operatively is…excellent…I’m very satisfied with them (laughs).* (Doctor 8, Maputo Central Hospital)

*Support of local and international institutions*. Clinicians reported that partnerships with international organisations and local institutions facilitate the treatment of paediatric injuries by bringing new and advanced knowledge and surgical supplies and help with specialised care, ambulances and laboratory tests.

*Facilitators we are ourselves, with the few resources we have…we have some friends from the United States, they have helped us a lot, they come once a year in August, they are friends of the surgeon who treats the paediatric traumas, they help us a lot with material and new dynamics, also work, they learn from us, and we also learn from them… I think this is the only facilitator we have.* (Nurse 5, Matola Provincial Hospital)

## Discussion

This study sheds light on the barriers and facilitators experienced by clinicians taking care of injured children in Maputo, Mozambique. It also provides insights that build from all levels of care, with a focus on a SSA resource-constrained setting. Not surprisingly, many challenges were identified, several internal to the unit and hospitals considered, but others related to the system including prehospital care.

Prehospital challenges were presented by many as a source of barriers to the provision of timely and quality care to injured children. This, in turn, reflects the rudimentary organisation of trauma services where the prehospital phase remains underdeveloped.^37^ Consequently, as seen in other resource-poor settings,^37 39^ delays in receiving timely care (due to, eg, the transport of patients to the hospital) are common and put children at extra risk, not least when they get transported from the injury site to the nearest care facility in vehicles inappropriately equipped and lacking equipment or trained people. Not surprisingly, many preventable deaths occur, with estimates showing that, in LMICs, up to 80% of injury deaths occur outside the hospital, ambulance access being, in the main, restricted to transport between hospitals.^40^

Besides the shortcomings derived from prehospital conditions, the study also reveals barriers in resources within hospitals, including equipment and medication that are lacking, malfunctioning or poorly distributed, for instance, essential medications, surgical supplies and equipment for diagnosis, monitoring or surgical interventions. These findings align with several (checklist based) studies from SSA.^8^,^1141^ Added to this is the fact that the resources in place are often ill-adapted to children’s specific needs/attributes. Consequently, they take more time to care for injured children, and this contributes to poor health outcomes due to the pressure of work time and overload. This is a finding common to studies from LMICs where resource allocation prioritises infectious diseases and pays less attention to epidemiological changes.^42^

Further, in all four hospitals, the adult emergency was the only place of admission for injured patients, although the three higher-level hospitals have paediatric emergency services, with paediatric emergency professionals. Inadequately organised infrastructure (eg, the layout itself, space and mixed populations) is an additional source of concern in paediatric trauma care.^17^

The lack of skills and knowledge in paediatric injury care and the absence of child-friendly resources may have several consequences that are not specific to clinicians from LMICs.^24^ One is that after stabilising the patient, most clinicians will refer the child to a specialised hospital. By doing so, additional delays in managing the patient occur, the risk of poorer outcomes rises, and specialised hospitals get overloaded.^18^ Alongside the lack of training, the shortage of specialists is also a critical issue in SSA, limiting the number of staff able to perform essential emergency care.^5 41 43 44^ Some countries, like Tanzania, may have staff trained in paediatric critical care but still lack adequate services and facilities.^24^ This finds an echo in the narrative of clinicians in remote areas who experience that their competence cannot be fully used because they work in resource-poor environments that do not allow them to put their skills into practice. To make things worse, transport to a more specialised hospital may be dependent on availability and priority setting and takes place in ambulances lacking resuscitation resources.^1 2^ Similar results were found in different studies in LMICs;^13 40 45^ a study from South Africa^46^ found that referring injured patients from district hospitals to higher levels of care delayed appropriate injury care and increased the risk of sepsis, urgent need for an intensive care unit, prolonged hospital stay and higher mortality.

To counteract some of the barriers identified and facilitate the delivery of quality care, clinicians of all levels of care highlighted the mentorship of local qualified teams and support of different institutions, local and international, as the most important facilitators of paediatric injury care. Interestingly, this echoes the findings of reviews on the mentorship of health personnel in LMICs.^43 47 48^ Also, a study in Mozambique^49^ reported that ‘north-south’ academic mentorship between two institutions facilitated staff training and improved the quality of paediatric surgical care.

Finally, the paediatric emergency department in the quaternary hospital is considered the most important facilitator. Clinicians at that level provide support and mentorship to those caring for children in critical condition, assisting both in diagnostic and care choices. Without this support, frontline clinicians experience stress and anxiety.^50^ In the same vein, the presence of a PICU supports clinicians, a finding that has been stressed in previous studies.^51^

### Strengths and limitations

One first strength of the study is that it presents the perspective of healthcare providers from several levels of care, dealing with paediatric injury in a resource-poor country, and highlights what emerges from their narrative. By doing so, it offers different perspectives on the local situation at each hospital and allows us to see how close the perceptions are within and across levels of care.

As there was an important consensus that emerged regarding the barriers experienced within and across levels, this gives an important insight into what, in Maputo city and province, prevents healthcare professionals from caring for injured children in the best possible way.

Whereas the perspective provided for tertiary and quaternary care is likely to give an adequate picture of the province, it might not be directly transferable to other parts of the country, where hospital services may not be equally developed, and the burden of child injury may be somewhat different. When it comes to lower-level hospitals, the information provided, although locally accurate and information, cannot be assumed to perfectly reflect all hospitals from similar levels in the province. As studies like this are rather uncommon, this is a meaningful contribution, strengthened by the fact that, in each of the four hospitals selected, all professionals accepted the invitation to participate, it was felt that they spoke openly, and the content of the interviews was rich.

An additional, potential limitation is a certain desirability bias that can have been introduced, since the first author and PI of the project is a known health professional in the hospitals of the province. It is of note however that she did not take part in the interview process. This can also have influenced the high level of participation obtained.

The study is silent regarding how important each barrier is within each hospital, as well as whether this picture would be the same if other—or different—hospitals had been involved. Even for hospitals from other levels of care, some discrepancy can be expected with other parts of the country, not least in remote areas. The study did not include the perspective of the leadership of the hospitals’ decision-makers; thus, it is difficult to determine an agenda for the improvement of child injury care.

To that end, it would probably be important to even conduct onsite resource assessments, using standardised tools, as was done a few years ago in the country’s largest hospitals.^15^ It is also of note that the study did not consider the perspective of the injured victims or their caregivers, which is undeniably an aspect that deserves greater attention.

Finally, as most of the studies evaluating emergency capacity in LMICs assess the availability of infrastructure and capacity to provide care by level^13^ but are not focused on paediatric injury care, this is the first study to help our understanding of paediatric injury care in Mozambique.

### Conclusion

From the clinicians’ perspective, barriers to paediatric injury care are often similar across hospitals and professional groups. Barriers include inadequate prehospital care. While lack of resources and poor infrastructure were—as expected—clearly emphasised, respondents identified a clear wish for knowledge- and competence-sharing.

## supplementary material

10.1136/bmjopen-2024-085270 online supplemental file 1

10.1136/bmjopen-2024-085270 online supplemental file 2

## Data Availability

All data relevant to the study are included in the article or uploaded as supplementary information.
