# Supplementary material for: Perspectives from clinicians from different levels of care in Maputo, Mozambique: qualitative study of the barriers to and facilitators of paediatric injury care in resource-poor hospital settings
Source: BMJ Open. 2024 Nov 24;14(11):e085270. doi: 10.1136/bmjopen-2024-085270 (PMC11590845; doi:10.1136/bmjopen-2024-085270)
Supplement: online supplemental file 1 [file bmjopen-14-11-s001.pdf]

Supplemental annex 1
ID do Participante: __|__|__|__|__|__|__|__|__|__|__|__|
Nome da unidade sanitária: ________________________

Estudo 3B

GUIÃO DE ENTREVISTA SEMI-ESTRUTURADA AOS CLÍNICOS DE SAÚDE

Protocolo com título: **"Traumatismo pediátrico agudo em um cenário africano de poucos recursos durante a pandemia de COVID-19: Percepções sobre epidemiologia de traumatismos e cuidados hospitalares de Moçambique". Versão 6.0. Data 05/09/2022.**

Obrigado por falar comigo **____** sobre a pesquisa "**Avaliação dos obstáculos e facilitadores para melhorar o atendimento aos traumatismos pediátricos identificados por clínicos da Província de Maputo em diferentes níveis de atendimento**". Este estudo tem como objectivos:

- Explorar os obstáculos e facilitadores vivenciados por clínicos de saúde relacionados com a qualidade na prestação de cuidados a pacientes pediátricos traumatizados;
- Explorar os obstáculos e facilitadores para melhorar o atendimento a traumatismos pediátricos identificados por clínicos de saúde na província de Maputo em diferentes níveis de atendimento.

Não precisa responder a nenhuma pergunta que não deseja ou que lhe deixe constrangido. Em caso de se sentir constrangido em responder, pode interromper a sua participação a qualquer momento. A sua participação nesta pesquisa é voluntária.

Gostaria de continuar?

[Se não, agradeça a pessoa e termine a entrevista]
[Se sim, continue com a entrevista]

Supplemental annex 1
ID do Participante: __|__|__|__|__|__|__|__|__|__|__|__|
Nome da unidade sanitária: ________________________

[FAÇA O CONSENTIMENTO INFORMADO]
DADOS SOCIODEMOGRÁFICOS

1. Género	□ Masculino (1)<br>□ Feminino (2)
2. Quantos anos você tem?	Idade completa ☐☐
3. Em que posição trabalha?	___________________<br>*(escolha entre as categorias de interesse)*
4. Quando é que você começou a trabalhar nessa posição?	_____ (ano)

Supplemental annex 1
ID do Participante: __|__|__|__|__|__|__|__|__|__|__|__|
Nome da unidade sanitária: ________________________

---

1. Agora gostaria que falasse sobre sua experiência com os serviços de atendimento ao trauma.
   Explore:
   a) Quais são as suas principais responsabilidades?
   b) No seu trabalho diário tem atendido crianças?
   Se NÃO, porquê não atende crianças? Quem é que atende? Onde essas crianças são atendidas? Se existe diferença no atendimento de trauma em adultos e crianças?

2. Há quanto tempo trata de crianças com traumatismos?
   Explore:
   a) Que tipo de traumatismos trata?
   b) Qual é a condição socioeconómica dos pacientes que atende?

3. Quais são as dificuldades que enfrenta no seu trabalho? E o que pode ser feito para que essas dificuldades sejam ultrapassadas?
   Explore:
   a) Quais são as dificuldades que encontra para atendimento adequado ao traumatismo pediátrico?
   b) Na sua opinião, qual o principal motivo dessas dificuldades (*falta de infra-estruturas adequadas, ferramentas cirúrgicas, suplementos médicos ou equipamentos necessários para cirurgias*)?

4. Como você se sente quando atende crianças com traumatismos graves?
   Explore:
   a) Os medos e as limitações que os clínicos têm durante o atendimento a pacientes com traumatismo pediátrico.

5. Quais são os facilitadores que encontra no serviço de urgência em relação ao atendimento a pacientes com traumatismo pediátrico?

6. O que acha da qualidade do tratamento prestado a pacientes com traumatismo pediátrico?

7. O que propõe para melhorar a qualidade da prestação de cuidados aos pacientes com traumatismo pediátrico?

8. Quais são os facilitadores que encontra no serviço de urgência em relação ao atendimento a pacientes com traumatismo pediátrico?

9. Há mais alguma coisa que gostaria de partilhar relativamente às barreiras e facilitadores no atendimento de pacientes com traumatismo pediátrico que ainda não tenhamos perguntado?

Muito obrigado pelo seu tempo!
