# Supplementary material for: Perspectives from clinicians from different levels of care in Maputo, Mozambique: qualitative study of the barriers to and facilitators of paediatric injury care in resource-poor hospital settings
Source: BMJ Open. 2024 Nov 24;14(11):e085270. doi: 10.1136/bmjopen-2024-085270 (PMC11590845; doi:10.1136/bmjopen-2024-085270)
Supplement: online supplemental file 2 [file bmjopen-14-11-s002.pdf]

**Supplemental annex 2**
**ID do Participante: __|__|__|__|__|__|__|__|__|__|__|__|**
**Nome da unidade sanitária: ________________________**

---

**Estudo 3B**

**TERMO DE CONSENTIMENTO INFORMADO AOS CLÍNICOS DE SAÚDE**

Protocolo com o título **"Traumatismo pediátrico aguda em um cenário africano de poucos recursos durante a pandemia de COVID-19: Percepções sobre epidemiologia de traumatismos e cuidados hospitalares de Moçambique". Versão 6.0. Data 05/09/2022.** Investigadora Principal: Vanda Amado, Faculdade de Medicina da Universidade Eduardo Mondlane e Instituto Karolinska, Estocolmo.

Obrigado por falar comigo _________________________ sobre a pesquisa "**Avaliação dos obstáculos e facilitadores para melhorar o atendimento aos traumatismos pediátricos identificados por clínicos da Província de Maputo em diferentes níveis de atendimento**". Este estudo tem como objectivos:

- Explorar os obstáculos e facilitadores vivenciados por clínicos de saúde relacionados com a qualidade na prestação de cuidados a pacientes pediátricos traumatizados;
- Explorar os obstáculos e facilitadores para melhorar o atendimento a traumatismos pediátricos identificados por clínicos de saúde na província de Maputo em diferentes níveis de atendimento.

O estudo é patrocinado pelo programa de Cooperação para pesquisa "UEM-Suécia" e financiado pelo SIDA-Swedish International Development Cooperation, Agency grant number 51140073.

O Objectivo deste estudo é avaliar as barreiras e facilitadores para melhorar o atendimento a lesões agudas pediátricas identificadas pelos clínicos hospitalares em Maputo. Foi selecionado para participar deste estudo porque o mesmo tem como grupo-alvo clínicos que prestam cuidados de saúde hospitalares a crianças com traumatismos agudos.

Meu nome é Vanda Nilza Sidónio Amado e estou a fazer uma pesquisa em traumatismos pediátricos (intoxicação, queimaduras, quedas, afogamentos, acidente de viação, violência domestica e/ou sexual) nos hospitais da Província de Maputo. A pesquisa tem como objectivo o melhoramento intra-hospitalar de assistência às crianças com trauma pediátrico agudo. Assim sendo, gostaríamos de entrevistá-lo/a sobre a sua percepção em relação a este tópico.

Gostaria de convidá-lo/a a participar no estudo. A sua participação é voluntária. Se decidir não participar, essa decisão não trará qualquer implicação negativa para si. Poderá desistir de participar no estudo sem necessidade de justificar a sua decisão e sofrer qualquer represália.

**Supplemental annex 2**
**ID do Participante: __|__|__|__|__|__|__|__|__|__|__|__|__|**
**Nome da unidade sanitária: ________________________**

---

Durante a sua participação no estudo, estarei disponível para em qualquer momento esclarecer as dúvidas que possa ter. Por favor, coloque todas as questões que tiver para que eu possa esclarecer.

Trata-se de uma entrevista, que será por mim conduzida ou pelos assistentes. A entrevista não levará mais que 60 minutos. A entrevista aborda aspectos sobre como melhorar a assistência de traumatismos pediátricos agudos. A entrevista é estritamente anónima e será gravada para posterior análise. Gostaria que autorizasse a gravação.

Para garantir confidencialidade, apenas a equipa de pesquisadores terá acesso a informação relativa a esta entrevista e a sua identidade. A sua participação é voluntária, e como prova de autorização para a realização da entrevista irei solicitá-lo/a para assinar a folha de consentimento informado.

Ao participar no estudo não correrá nenhum risco. Apesar de se requerer a sua assinatura que prove que aceitou voluntariamente participar na entrevista, este documento será imediatamente separado dos dados da entrevista. O seu nome não aparecerá durante a análise dos dados, nem na apresentação dos resultados. Toda a informação que fornecer será apenas usada para fins científicos, é confidencial e apenas a investigadora principal terá acesso à mesma. Toda a informação da entrevista será analisada de forma anónima.

A participação no estudo não terá nenhum benefício directo, mas irá ajudar a compreender os obstáculos e facilitadores para a assistência ao traumatismo pediátrico agudo.

O presente estudo foi revisto e aprovado pelo Comité Institucional de Bioética em Saúde da Faculdade de Medicina/Hospital Central de Maputo-CIBS FM&HCM-Faculdade de Medicina, Av. Salvador Allende, 702, Maputo.

Enquanto o estudo estiver a decorrer a investigadora principal Vanda Nilza Sidónio Amado, estará disponível para esclarecer qualquer dúvida relacionada com a investigação, podendo contactá-la através do número de telefone 876269830 ou ao Comité Institucional de Bioética em Saúde da Faculdade de Medicina/Hospital Central de Maputo-CIBS FM&HCM-Faculdade de Medicina, Faculdade de Medicina, Av. Salvador Allende nº702, telefone: 21428076 www.cibs.uem.mz cujo contacto telefónico da presidente é (+258) 823992590 e do vice-presidente é (+258) 846073868.

**Supplemental annex 2**
**ID do Participante: __|__|__|__|__|__|__|__|__|__|__|__|**
**Nome da unidade sanitária: ________________________**

---

Eu (NOME DO PARTICIPANTE),_______________________________________________,
Li e ouvi o texto acima e entendi a natureza e objectivo do estudo do qual fui convidado a participar. O pesquisador me explicou sobre os riscos e benefícios do estudo. Eu entendi que sou livre para interromper minha participação no estudo a qualquer momento sem justificar minha decisão e sem que essa decisão me prejudique de qualquer maneira. Concordo voluntariamente em participar desse estudo e tenho a concordância do coordenador da instituição para participar.

________________________________________________________
Nome do participante (em maiúsculas)

________________________________________________________
Assinatura do participante
Data: _______/_______/_______ Hora: _____:_____

________________________________________________________
Nome (em maiúscula) da pessoa que realizou a explicação do consentimento

________________________________________________________
Assinatura da pessoa que realizou a explicação do consentimento
Data: _______/_______/_______ Hora: _____:_____
