# Supplementary material for: Perspectives from clinicians from different levels of care in Maputo, Mozambique: qualitative study of the barriers to and facilitators of paediatric injury care in resource-poor hospital settings
Source: BMJ Open. 2024 Nov 24;14(11):e085270. doi: 10.1136/bmjopen-2024-085270 (PMC11590845; doi:10.1136/bmjopen-2024-085270)
Supplement: Supplementary file 3 [file bmjopen-2024-085270.reviewer_comments.pdf]

PEER REVIEW HISTORY

BMJ Open publishes all reviews undertaken for accepted manuscripts. Reviewers are asked to complete a checklist review form and are provided with free text boxes to elaborate on their assessment. These free text comments are reproduced below.

ARTICLE DETAILS

Title (Provisional)

Qualitative study of the barriers to and facilitators of paediatric injury care in poor-resource hospital settings: Perspectives from clinicians from different levels of care in Maputo, Mozambique

Authors

Amado, Vanda; Zandamela, Americo; Couto, Maria Tereza; Wallis, Lee A; LAFLAMME, Lucie

---

VERSION 1 - REVIEW

**Reviewer**	**1**
**Name**	**Botelho, Fabio**
**Affiliation**	**McGill University**
**Date**	**22-Feb-2024**
**COI**	**None**

I express my positive appraisal of the study titled "Barriers and facilitators to paediatric injury care in poor-resource hospital settings: Perspectives from clinicians from different levels of care in Maputo Mozambique." The clarity of the qualitative analysis conducted are commendable, effectively capturing the nuanced perspectives of the participants.

I appreciate the study's straightforward approach, adhering strictly to its qualitative nature without overextension. My minor suggestions for improvement include verifying the potentially mistyped density number "3 628,2hab./m2," which might more accurately be "km2" instead of "m2." Additionally, it would be beneficial to list the specialists in Table 2 for the Maputo General Hospital and to add a corresponding legend for clarity.

Overall, the study is a valuable contribution to the field, highlighting critical areas for intervention and improvement in pediatric trauma care within resource-limited settings. I commend the authors for their dedication to this important work, and I hope to read future studies addressing the identified barriers. Such research is vital for developing replicable models of care improvement in similar contexts worldwide. Congratulations on leading this insightful study.

**Reviewer**	**2**
**Name**	**Nabwera, Helen**
**Affiliation**	**Liverpool School of Tropical Medicine, International Public Health**
**Date**	**08-May-2024**
**COI**	**None**

Barriers and facilitators to paediatric injury care in poor-resource hospital settings: Perspectives from clinicians from different levels of care in Maputo, Mozambique bmjopen-2024-085270

Overall comments:

This manuscript addresses a key child health priority – the slow progress in reducing child morbidity and mortality in low-and middle-income countries from injuries/trauma. The exploration of health care worker perspectives on where the gaps lie and what the possible solutions are in Mozambique, is a worthwhile study to inform health system strengthening for child trauma/injury prevention and management. It was useful to incorporate the section on reflexivity – a key component of collecting and interpreting qualitative data.

However, there are number of areas that need to be addressed in order to strengthen it. There were several grammatical errors and spelling mistakes that need to be addressed. In addition, the structure of the Methods section needs to be revised as there are key details missing. Finally, most sections could be written more succinctly. I have made some suggestions that I hope are helpful.

Abstract

Page 1 Line 59: Providing care for injured children faces multiple challenges in resource-poor settings.

Please amend to "Providing care for injured children is challenging in resource-poor settings."

Page 2 Line 8: Methods: In qualitative research purposive sampling is done for participants and not of hospitals as you have stated here. Please revise.

Key messages

Page 2, line 39 (bullet point 3): please revise this sentence that is grammatically incorrect.

Strengths

Page 2, line 50 (bullet point 2): for qualitative research it is not the number of interviews that strengthens the study, it is the depth and breadth of information that is collected, being

able to achieve “data saturation” and address your research questions. Please revise this statement.

Limitations

Bullet points 1 and 3 are stating the same thing.

Spelling error “weather” instead of “whether” bullet point 4

Introduction

Page 3, line 2: … “Injuries are the leading cause of death…” Please start this sentence with “globally” – otherwise it is incomplete.

There are sections that are well-written with useful arguments and justifications. However, there is also repetition. To present this more succinctly, I would suggest that you write paragraph one more succinctly and delete paragraph 2.

The study objectives are well-described.

Methods

• Study design

o This is too brief and doesn’t provide the level of information required for a scientific paper. This needs to be better described with some references to justify the approach.

• Study setting and selection of hospitals

o This should be written more succinctly.

o Again, it is unusual to use the term purposive sampling for the hospitals. This should refer to the participants and provide a justification for this.

• Study participants

o The first paragraph should be moved to an additional section “Ethical consideration”. Here consent can also be described.

• Please include a justification for the sample size.

Data analysis

This is described sufficiently.

Results

Table 2 suggests that structured observations if the equipment in the hospitals or a questionnaire was administered to collect these data. There are not details of this in the

Methods. Please review this.

The results are well described with quotes from different participants incorporated.

Discussion

The discussion addresses the key findings, their interpretation and how the current literature supports these findings. The authors also reflect on the limitation of the observational nature of their analysis.

Further reflections on the strengths/limitations of this study would be important.

Conclusion

This is succinct and captures the key messages.

VERSION 1 - AUTHOR RESPONSE

**Reviewer 1:** Dr. Fabio Botelho, McGill University	
My minor suggestions for improvement include verifying the potentially mistyped density number "3 628,2hab./m2," which might more accurately be "km2" instead of "m2."	Mistyped density corrected.
Additionally, it would be beneficial to list the specialists in Table 2 for the Maputo General Hospital and to add a corresponding legend for clarity.	Table 2 has been revised to incorporate those suggestions and to improve clarity about the staff categories and equipment across hospitals.<br><br>See results page 8
**Reviewer 2:** Dr. Helen Nabwera, Liverpool School of Tropical Medicine	
However, there are a number of areas that need to be addressed in order to strengthen it.<br>There were several grammatical errors and spelling mistakes that need to be addressed.<br><br>In addition, the structure of the Methods section needs to be revised as there are key details missing.<br><br>Finally, most sections could be written more succinctly. I have made some suggestions that I hope are helpful.	Gramar and typos corrected using the software proposed by the journal<br><br>Revision done in accordance with the COREQ guidelines, implying some sub-sections were added

Abstract<br>Page 1 Line 59: Providing care for injured children faces multiple challenges in resource-poor settings.<br>Please amend to "Providing care for injured children is challenging in resource-poor settings."	Done.
Page 2 Line 8: Methods: In qualitative research purposive sampling is done for participants and not of hospitals as you have stated here. Please revise	Done. See abstract page 2
Key messages<br>Page 2, line 39 (bullet point 3): please revise this sentence that is grammatically incorrect.	Please note that the key messages have been revised to comply to the journal requirement.<br>Point taken on number of interviews not being a strength in qualitative research.
Strengths<br>Page 2, line 50 (bullet point 2): for qualitative research it is not the number of interviews that strengthens the study, it is the depth and breadth of information that is collected, being able to achieve "data saturation" and address your research questions. Please revise this statement.	
Limitations<br>Bullet points 1 and 3 are stating the same thing.<br>Spelling error "weather" instead of "whether" bullet point 4	See comment above on revision of key messages.
Introduction<br>Page 3, line 2: … "Injuries are the leading cause of death…" Please start this sentence with "globally" – otherwise it is incomplete.<br>There are sections that are well-written with useful arguments and justifications. However, there is also	Correction made.<br><br>Please note that the section has received major revisions. Section revisited accordingly.

repetition. To present this more succinctly, I would suggest that you write paragraph one more succinctly and delete paragraph 2.<br>The study objectives are well-described	
• Study design<br>o This is too brief and doesn't provide the level of information required for a scientific paper. This needs to be better described with some references to justify the approach.<br>• Study setting and selection of hospitals<br>o This should be written more succinctly.<br>o Again, it is unusual to use the term purposive sampling for the hospitals. This should refer to the participants and provide a justification for this.<br>• Study participants<br>o The first paragraph should be moved to an additional section "Ethical consideration". Here consent can also be described.<br>• Please include a justification for the sample size.<br>Data analysis<br>This is described sufficiently.	The section has been restructured in accordance with the COREQ guidelines, and all comments and suggestions mentioned have been addressed<br><br><br><br>See methods page 4-6
Results<br>Table 2 suggests that structured observations if the equipment in the hospitals or a questionnaire was administered to collect these data. There are not details of this in the Methods. Please review this.<br>The results are well described with quotes from different participants incorporated.	Table 2 has been reorganised to incorporate those suggestions and is better explained in the.<br><br>See methods page 6and results page 8.

Discussion<br>The discussion addresses the key findings, their interpretation and how the current literature supports these findings. The authors also reflect on the limitation of the observational nature of their analysis.<br>Further reflections on the strengths/limitations of this study would be important.	Further Strengths and limitations incorporate in the 5 bullets about strengths and limitations and in the discussions.<br><br>See discussion page 16-17.

VERSION 2 - REVIEW

Reviewer 2

Name Nabwera, Helen

Affiliation Liverpool School of Tropical Medicine, International Public Health

Date 31-Jul-2024

COI None.

Barriers and facilitators to paediatric injury care in poor-resource hospital settings: Perspectives from clinicians from different levels of care in Maputo, Mozambique bmjopen-2024-085270

Overall

There is a marked improvement in the grammar in this manuscript. However, the sentences are long and complex with limited punctuation, making them challenging to comprehend.

The Methods sections needs to be reorganised. Although the content is much improved, it does not follow the standard format. Please review this.

Further amendments on the Results section are also required.

Introduction

Please use correct sub-Saharan - the "sub" should not be start with a capital "S"

Methods

Participant selection

The health clinicians from Maputo Province Hospitals were purposively selected (29,30) to better identify and selecting health providers with a range of knowledge and experience in paediatric injury

care that would contribute to identify and obtain information related to barriers and facilitators to improve paediatric injury care.

This sentence is long with grammatical errors. Please break it up into 2 sentences and address the grammatical errors.

The rest if this section would benefit from improved punctuation of the complex sentences.

Study setting and selection of hospitals

In this study, a convenience sampling ensuring a mix of all levels of care including district and referral hospital was done, focused on Maputo.

Unfortunately, I disagree – this is not "convenience sampling." It would be more accurate to outline the rationale of how the hospitals were selected and avoid giving this process an incorrect name.

This sub-section should come first in the Methods section otherwise it will be difficult for the reader to understand the context.

Please delete the sub-title: "Analysis and Results"

Results

Table 2: *Hospital specify capacity limitations reported by the interviewers, from the quarterly hospital to secondary care.*

Please correct the grammatical errors here. Also, the participants reporting this are the "interviewees" and not the "interviewers" as stated.

Quotes: as all the hospitals had more than one doctor and more that one nurse, it is important that the quotes reflect this. Otherwise, the reader cannot tell whether these quotes are from the same doctors and nurses in each of the hospitals.

Study strengths and limitations

Please review the punctuation of the long and complex sentences.

VERSION 2 - AUTHOR RESPONSE

**Reviewer 2: Dr. Helen Nabwera, Liverpool School of tropical Medicine**	
Overall	
There is a marked improvement in the grammar in this manuscript. However, the sentences are long and complex with limited punctuation, making them challenging to comprehend.	Revision of the sentences and punctuation were taken core by a native English-speaking co-author.
The Methods sections needs to be reorganised. Although the content is much improved, it does not follow the standard format. Please review this.	Methods section reorganized following the BMJ open authors submission guidelines recommendations – https://bmjopen.bmj.com/pages/authors?_gl=1*bhjfag*_ga*Njg5MzA5NjI5LjE2Nj

Further amendments on the Results section are also required	E4NjQ3MTI.*_ga_EXTSVLH45V*MTcyNDEyMzk1MS4zNS4xLjE3MjQxMjM5NzQuMC4wLjM2NDQ1NDAzNw..#research and accordance with the COREQ guidelines
Introduction	
Please use correct sub-Saharan - the “sub” should not be start with a capital “S”	Correction done and whole article cross-checked for consistency.<br><br>See page 3
Methods	
Participant selection<br><br>The health clinicians from Maputo Province Hospitals were purposively selected (29,30) to better identify and selecting health providers with a range of knowledge and experience in paediatric injury care that would contribute to identify and obtain information related to barriers and facilitators to improve paediatric injury care.<br>This sentence is long with grammatical errors. Please break it up into 2 sentences and address the grammatical errors.<br>The rest if this section would benefit from improved punctuation of the complex sentences.	Sentence split and revised for grammatical errors.<br><br>See page 5
Study setting and selection of hospitals<br><br>In this study, a convenience sampling ensuring a mix of all levels of care including district and referral hospital was done, focused on Maputo.<br><br>Unfortunately, I disagree – this is not “convenience sampling.” It would be more accurate to outline the rationale of how the hospitals were selected and avoid giving this process an incorrect name.	Done. Text moved up, following study design, as BMJ open recommendation (see above). See page 4.

This sub-section should come first in the Methods section otherwise it will be difficult for the reader to understand the context.	
Please delete the sub-title: "Analysis and Results"	Done, see page 7
**Results**	
Table 2: Hospital specify capacity limitations reported by the interviewers, from the quarterly hospital to secondary care.<br>Please correct the grammatical errors in this legend. Also, the participants reporting this are the "interviewees" and not the "interviewers" as stated.	Changes done. Apology for the confusion.<br>See page 8
Quotes: as all the hospitals had more than one doctor and more than one nurse, it is important that the quotes reflect this. Otherwise, the reader cannot tell whether these quotes are from the same doctors and nurses in each of the hospitals.	Done. Doctors and nurses quoted were attributed numbers. see page 10-14.
**Study strengths and limitation**	
Please review the punctuation of the long and complex sentences.	Done see pages 16 and 17

VERSION 3 - REVIEW

**Reviewer**	**2**
**Name**	**Nabwera, Helen**
**Affiliation**	**Liverpool School of Tropical Medicine, International Public Health**
**Date**	**24-Sep-2024**
**COI**	

This revised manuscript reads a lot better. Well done to the authors. However, there are a few gaps in the Methods section that need to be addressed. Please find these below.

Methods

Participant selection

We aimed to interview around 10 clinicians per hospital, covering the most representative professional categories (surgeons, paediatricians, nurses, and technicians). We continued until thematic saturation was achieved.

Please explain what you mean by "thematic saturation," its relevance to this study and provide a reference for it. This should ideally be outlined under a sub-section “Sample size”. Or the authors can add this to the above sub-section i.e. “Participant selection and sample size”

Data analysis

There is no mention on how the “observations” that have now been added to the Methods section were incorporated into the analysis. Please add these details.

Also, kindly proof read it again, as there are still some typing errors.

VERSION 3 - AUTHOR RESPONSE

Reviewer(s)' Comments	Authors’ response
**Reviewer 2: Dr. Helen Nabwera, Liverpool School of tropical Medicine**	
Participant selection<br><br>We aimed to interview around 10 clinicians per hospital, covering the most representative professional categories (surgeons, paediatricians, nurses, and technicians). We continued until thematic saturation was achieved.<br><br>Please explain what you mean by "thematic saturation," its relevance to this study and provide a reference for it. This should ideally be outlined under a sub-section “Sample size”. Or the authors can add this to the above sub-section i.e. “Participant selection and sample size”	Information provided in the sub-section Participants selection. See page 5

Data analysis<br>There is no mention on how the "observations" that have now been added to the Methods section were incorporated into the analysis. Please add these details.<br><br>Also, kindly proof-read it again, as there are still some typing errors.	Information provided. See page 6<br><br>Proof-reading done by a specialist.
